# Trajectory pattern of serially measured acute kidney injury biomarkers in critically ill patients: a prospective observational study

**DOI:** 10.1186/s13613-024-01328-9

**Published:** 2024-06-06

**Authors:** Ryohei Horie, Naoki Hayase, Toshifumi Asada, Miyuki Yamamoto, Takehiro Matsubara, Kent Doi

**Affiliations:** Department of Emergency and Critical Care Medicine, The University of Tokyo Hospital, 7-3-1 Hongo, Bunkyo-Ku, Tokyo, 113-8655 Japan

**Keywords:** Acute kidney injury, Biomarker, Trajectory, Neutrophil gelatinase-associated lipocalin, Liver-type fatty acid-binding protein, Major adverse kidney event, Group-based trajectory modeling

## Abstract

**Background:**

The clinical value of the trajectory of temporal changes in acute kidney injury (AKI) biomarkers has not been well established among intensive care unit (ICU) patients.

**Methods:**

This is a single-center, prospective observational study, performed at a mixed ICU in a teaching medical institute in Tokyo, Japan. Adult ICU patients with an arterial line and urethral catheter were enrolled from September 2014 to March 2015. Patients who stayed in the ICU for less than 48 h and patients with known end-stage renal disease were excluded from the study. Blood and urine samples were collected for measurement of AKI biomarkers at 0, 12, 24, and 48 h after ICU admission. The primary outcome was major adverse kidney events (MAKE) at discharge, defined as a composite of death, dialysis dependency, and persistent loss of kidney function (≥ 25% decline in eGFR).

**Results:**

The study included 156 patients. Serum creatinine-based estimated glomerular filtration rate (eGFR), plasma neutrophil gelatinase-associated lipocalin (NGAL), and urinary liver-type fatty acid-binding protein (uL-FABP) were serially measured and each variable was classified into three groups based on group-based trajectory modeling analysis. While the trajectory curves moved parallel to each other (i.e., “low,” “middle,” and “high”) for eGFR and plasma NGAL, the uL-FABP curves showed distinct trajectory patterns and moved in different directions (“low and constant,” “high and exponential decrease,” and “high and exponential increase”). These trajectory patterns were significantly associated with MAKE. MAKE occurred in 16 (18%), 16 (40%), and 9 (100%) patients in the “low and constant,” “high and exponential decrease,” and “high and exponential increase” groups, respectively, based on uL-FABP levels (p-value < 0.001). The initial value and the 12-h change in uL-FABP were both significantly associated with MAKE, even after adjusting for eGFR [Odds ratio (95% confidence interval): 1.45 (1.17–1.83) and 1.43 (1.12–1.88) for increase of initial value and 12-h change of log-transformed uL-FABP by 1 point, respectively].

**Conclusions:**

Trajectory pattern of serially measured urinary L-FABP was significantly associated with MAKE in ICU patients.

**Supplementary Information:**

The online version contains supplementary material available at 10.1186/s13613-024-01328-9.

## Background

Acute kidney injury (AKI) is a common problem in various clinical settings, including intensive care units (ICUs) [1–5]. AKI is associated with poor clinical outcomes, including increased mortality, renal replacement therapy (RRT) dependence, and progression to chronic kidney disease (CKD) [2, 3, 5–8]. Although substantial efforts have been made to accurately diagnose AKI early and improve outcomes [4, 9], challenges in these areas still exist. The current standard diagnostic criteria for AKI are based on the Kidney Disease: Improving Global Outcomes (KDIGO) criteria; AKI is diagnosed based on serum creatinine (sCr) and urine output (UOP) levels [10]. However, sCr and UOP are indices of kidney function, rather than kidney damage, and may not directly reflect the pathology of AKI [9].

To address limitations in diagnosing AKI, various biomarkers for direct assessment of renal injury have been proposed [11, 12]. Neutrophil gelatinase-associated lipocalin (NGAL) and liver-type fatty acid-binding protein (L-FABP) are direct biomarkers of kidney damage. NGAL and L-FABP originate mainly in the distal and proximal tubules, respectively, and detect early-stage AKI [9]. For example, elevated urine or plasma NGAL levels are associated with higher mortality rates among critically ill patients, even without sCr elevation [13]. Perioperative urine L-FABP (uL-FABP) predicts AKI development before sCr elevation among patients undergoing abdominal aneurysm repairs [14]. In addition to early detection of AKI, clinical studies reported promising results for these novel biomarkers in the prognosis of critically ill patients [13, 15–24]. Recently, a new framework of AKI classification has been proposed, in which both functional and damage biomarkers are combined [25].

The associations of most AKI biomarkers with clinical outcomes were examined at single time points. Only a few studies reported the impact of serial measurements [26–29], and the patterns of chronological changes were not assessed. In this study, the trajectory patterns of plasma NGAL and uL-FABP were investigated using group-based trajectory modeling [30–32]. The objective of this study was to evaluate the association of the trajectory patterns of AKI biomarkers with major adverse kidney events (MAKE), defined as the composite outcome of death, new dialysis, and worsening renal function [33].

## Methods

### Study design

The aim of this study was to evaluate the association of the trajectory patterns of AKI biomarkers with MAKE among critically ill patients. A single-center, prospective observational study was performed in a mixed ICU at the University of Tokyo Hospital, Tokyo, Japan. The study protocol was approved by the Research Ethics Committee of the Faculty of Medicine of the University of Tokyo (Approval No. 2810-13; title: “Establishment of blood and urinary biomarker for acute kidney injury” on December 7, 2009). Written informed consent was obtained from the patient or surrogate decision maker. All procedures were followed in accordance with the ethical standards of the Research Ethics Committee of the Faculty of Medicine of the University of Tokyo and with the Helsinki Declaration of 1975.

### Patients

Adult patients admitted to the ICU from September 2014 to March 2015 were screened for enrollment. The inclusion criteria were: (1) patients with an arterial line and urethral catheter for monitoring blood pressure and urine output so serial blood and urine tests could be performed with the existing catheters and (2) written informed consent was signed by the patient or the surrogate decision maker. Patients meeting the following criteria were excluded: (1) patients who were discharged from the ICU within 48 h of ICU admission and (2) patients who had a known diagnosis of end-stage renal disease (ESRD) before admission.

### Measurements

Upon ICU admission, patient demographics were obtained from the electronic medical records. The patients underwent serial blood and urine tests, including sCr for estimated glomerular filtration rate (eGFR), plasma NGAL, and uL-FABP, at 0, 12, 24, and 48 h after ICU admission. Serum creatinine levels were measured at the central laboratory of our hospital using the enzymatic method with LABOSPECT 008 α (Hitachi High-Tech®, reference ranges: 0.65–1.07 mg/dL for males and 0.46–0.79 mg/dL for females). The eGFRs were calculated based on the Modified Diet Renal Disease (MDRD) equation for Japanese patients [34]. Plasma NGAL was determined using the Triage® NGAL Device (Alere Medical, Inc., San Diego, CA, USA). Urinary L-FABP was measured using an enzyme-linked immunosorbent assay (Human L-FABP Assay Kit; CMIC, Tokyo, Japan). The uL-FABP values were normalized against urinary creatinine levels prior to analysis.

### Outcomes

The primary outcome was MAKE at hospital discharge, defined as a composite of death, dialysis dependency, and persistent loss of kidney function (≥ 25% decline in eGFR) [33]. The secondary outcomes included hospital death, the presence and the stage of AKI on day 7 after ICU admission, and RRT requirement during hospitalization. Sex, age, surgical intervention, acute physiology and chronic health evaluation (APACHE) II score, presence of CKD, sepsis, shock, and urinary tract infection were treated as potential confounders. The diagnosis of AKI was defined as a sCr increase from baseline of more than 0.3 mg/dL or 50% within 48 h or 7 days, respectively, based on the KDIGO guideline [10]. The baseline sCr and eGFR were defined as the last outpatient measurements within 6 months before ICU admission. For patients without an outpatient measurement, the baseline sCr was defined as the lowest value of admission sCr, discharge sCr, or sCr corresponding with an eGFR of 75 mL/min/1.73 m^2^ using the MDRD equation.

### Statistical analysis

R ver 4.1.0 was used for statistical analyses and p-values < 0.05 were considered significant. Plasma NGAL and uL-FABP were log-transformed prior to the analysis, due to their marked skewness in distribution.

The trajectory patterns were analyzed by performing group-based trajectory modeling for eGFR, log (NGAL), and log (uL-FABP). Group-based trajectory modeling is a statistical method which allows to categorize a group of patients into subclasses based on the trajectory patterns of a continuous variable changing over time (More details can be found in supplemental document). The trajectory patterns were modeled to follow the quadratic function of time in hours after ICU admission. Patients who had more than two missing measurements (out of all four measurements) in the variable of interest were excluded from the model. An extreme outlier was also excluded when it was necessary to generate meaningful trajectory models. Group-based trajectory modeling was performed using the lcmm package (ver 1.9.4), by assuming no random effects [31, 32]. The appropriate number of trajectory classes was selected based on the combination of the Bayesian information criterion, the number of patients in each class (at least 3% of the patients in the smallest class was considered the minimal requirement), and the model interpretability. The model performances were assessed using the average of the posterior probability assignment and the relative entropy. In this study, the models of three trajectory classes and those with four trajectory classes seemed appropriate. From the model interpretability stand point, 3-class models were selected and analyzed. Further statistical details of the trajectory analysis were summarized in supplemental document. The classes in the selected model and the outcomes were compared using Fisher’s exact tests.

A conventional statistical approach using a multivariate logistic regression model was also conducted. Multivariate logistic regression models to predict MAKE with different sets of predictor variables were created. The initial value (time0) and the changes between time 0 and 12 h of ICU stay (delta12) were used for eGFR, log (NGAL), and log (uL-FABP). For model comparison, the continuous net reclassification improvement (cNRI) and the integrated discrimination improvement (IDI) were calculated using the PredictABEL package (ver 1.2.4). After the best model was selected based on the cNRI and IDI [the model included time0 and delta12 for eGFR and log (uL-FABP)], the integrated uL-FABP index (FABPi) was defined as the fitted value by the model. The cutoff of FABPi was determined using the Youden index. Patients were divided into “positive” and “negative” groups based on the FABPi cutoff. MAKE were compared in patients with “positive” and “negative” FABPi after balancing the potential confounders (sex, age, surgical patients, acute physiology and chronic health evaluation [APACHE] II score, presence of CKD, sepsis, shock, and urinary tract infection) using inverse probability weighting (IPW). For evaluation of the clinical impact, the odds ratios with 95% confidence intervals were calculated based on the cluster-robust standard error using the lmtest (ver 0.9.40) and sandwich (ver 3.0.2) R packages. Patients with missing variables were excluded from the models requiring those specific missing variables.

## Results

### Patient characteristics

**Fig. 1:**
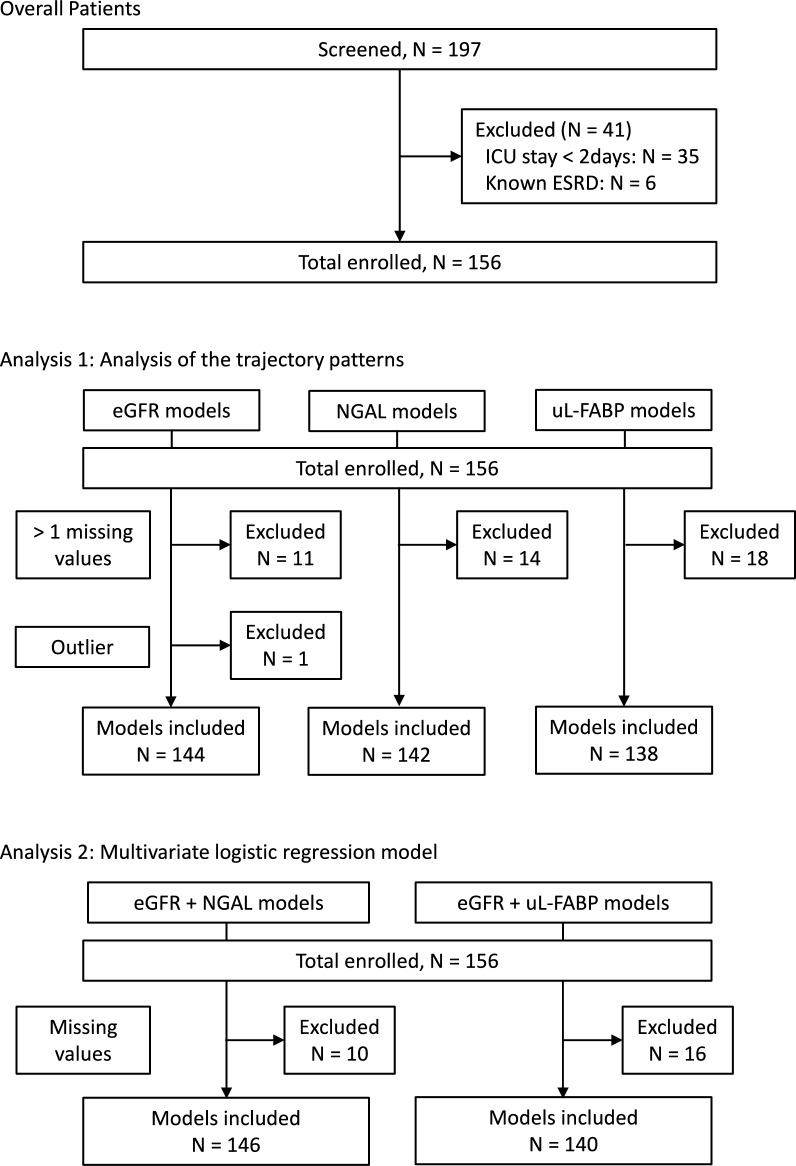
Study design and statistical model development. After patient enrollment, two types of analyses were performed, i.e., analysis of the trajectory patterns and multivariate logistic regression model. One patient (outlier) was excluded from the analysis of the trajectory patterns for eGFR, due to extremely high values (295.6, 347.0, 391.9, and 328.1 mL/min/1.73 m^2^ at time 0, 12, 24, and 48 h after ICU admission, respectively). *ESRD* end-stage renal disease, *eGFR* estimated glomerular filtration rate, *uL-FABP* urinary liver-type fatty acid-binding protein, *ICU* intensive care unit, *NGAL* neutrophil gelatinase-associated lipocalin

**Fig. 2:**
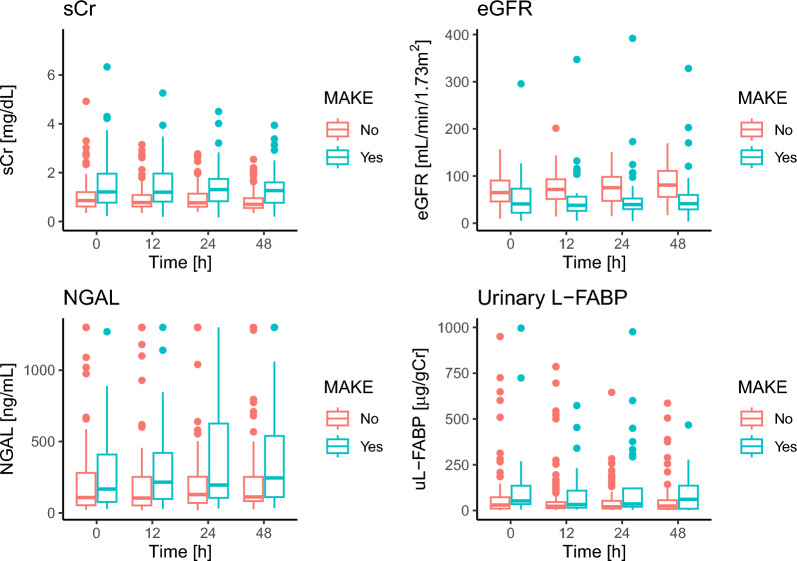
Renal function and AKI biomarkers at different time points. Patients who developed MAKE tended to have higher sCr, lower eGFR, higher plasma NGAL, and higher uL-FABP, as compared to those without MAKE. *eGFR* estimated glomerular filtration rate, *uL-FABP* urinary liver-type fatty acid-binding protein, *MAKE* major adverse kidney events at discharge, *NGAL* neutrophil gelatinase-associated lipocalin, *sCr* serum creatinine

During the study period, 197 patients were screened, of which 156 patients were enrolled. The study enrollment process and the number of excluded patients from each statistical model was shown in Fig. 1. The basic patient characteristics are shown in Table 1. The median age of the enrolled patients was 65-year-old with interquartile range (IQR) of 55- to 75-year-old, and 96 patients (62%) were male. MAKE occurred in 44 patients (28%). Stage 3 AKI was more common among those with MAKE. Patients with MAKE tended to be more severely ill than patients without MAKE, based on APACHE II and sequential organ failure assessment (SOFA) scores. The rates of sepsis, shock, UTI, surgery, and CKD did not show statistically significant differences between those with and without MAKE. As expected, patients with MAKE tended to have higher sCr, lower eGFR, higher plasma NGAL, and higher uL-FABP at ICU admission (Table 1 and Fig. 2). Marked skewness was observed in the distributions of plasma NGAL and uL-FABP; therefore, these parameters were log-transformed for further statistical analyses (Supplemental Fig. 1).

**Table 1:** Patient characteristics

Variable	Overall N = 156	MAKE ( +) n = 44	MAKE (−) n = 112	p-value
Age, y	65 (55, 75)	68 (57, 79)	64 (53, 73)	0.15
Sex, male	96 (62)	26 (59)	70 (62)	0.7
Death	22 (14)	22 (50)	0 (0)	< 0.001
ICU length of stay	5 (3, 9)	7 (3, 12)	5 (3, 8)	0.065
Hospital length of stay	44 (17, 80)	44 (13, 84)	44 (19, 78)	0.8
Day1 AKI				< 0.001
No AKI	85 (54)	16 (36)	69 (62)	
Stage 1	31 (20)	9 (20)	22 (20)	
Stage 2	18 (12)	5 (11)	13 (12)	
Stage 3	22 (14)	14 (32)	8 (7.1)	
Day7 AKI				< 0.001
No AKI	111 (74)	15 (38)	96 (86)	
Stage 1	20 (13)	9 (23)	11 (9.8)	
Stage 2	8 (5.3)	6 (15)	2 (1.8)	
Stage 3	12 (7.9)	9 (23)	3 (2.7)	
RRT need	17 (11)	13 (30)	4 (3.6)	< 0.001
Sepsis	20 (13)	7 (16)	13 (12)	0.5
Shock	24 (15)	8 (18)	16 (14)	0.5
UTI	6 (3.8)	3 (6.8)	3 (2.7)	0.4
Surgery	66 (42)	17 (39)	49 (44)	0.6
CKD	33 (21)	10 (23)	23 (21)	0.8
APACHE II score	16 (12, 22)	20 (14, 25)	15 (12, 21)	0.004
SOFA score (day1)	6 (4, 9)	8 (6, 10)	5 (3, 8)	< 0.001
sCr, mg/dL				
0 h	0.92 (0.63, 1.37)	1.22 (0.77, 2.09)	0.86 (0.62, 1.20)	0.002
12 h	0.86 (0.63, 1.30)	1.21 (0.82, 2.00)	0.78 (0.62, 1.08)	< 0.001
24 h	0.85 (0.62, 1.40)	1.32 (0.85, 1.85)	0.77 (0.60, 1.14)	< 0.001
48 h	0.78 (0.57, 1.26)	1.27 (0.77, 1.63)	0.70 (0.55, 0.96)	< 0.001
eGFR, mL/min/1.73m^2^				
0 h	58 (39, 86)	41 (22, 73)	65 (46, 91)	< 0.001
12 h	60 (40, 89)	38 (26, 56)	72 (51, 93)	< 0.001
24 h	60 (39, 92)	40 (30, 52)	75 (47, 98)	< 0.001
48 h	68 (41, 102)	41 (29, 60)	81 (56, 111)	< 0.001
NGAL, ng/mL				
0 h	112 (58, 320)	168 (77, 409)	108 (54, 281)	0.029
12 h	142 (66, 316)	216 (98, 421)	104 (52, 252)	0.007
24 h	137 (78, 357)	195 (106, 626)	128 (70, 253)	0.009
48 h	139 (85, 363)	246 (111, 540)	112 (83, 253)	0.010
uL-FABP, μg/gCr				
0 h	48 (14, 216)	130 (42, 1,038)	38 (12, 118)	< 0.001
12 h	28 (14, 124)	88 (21, 1,090)	25 (13, 52)	< 0.001
24 h	28 (12, 100)	72 (23, 394)	21 (11, 55)	< 0.001
48 h	35 (10, 85)	68 (14, 278)	25 (9, 56)	< 0.001

*MAKE* major adverse kidney events at hospital discharge, *ICU* intensive care unit, *AKI* acute kidney injury, *RRT* renal replacement therapy, *UTI* urinary tract infection, *CKD* chronic kidney disease, *APACHE II score* acute physiology and chronic health evaluation II score, *SOFA score* sequential organ failure assessment score, *sCr* serum creatinine, *eGFR* estimated glomerular filtration rate, *NGAL* neutrophil gelatinase-associated lipocalin, *uL-FABP* urinary liver-type fatty acid-binding protein. Continuous variables are shown in median (interquartile range) and categorical variables are shown in count (percentage). Wilcoxon rank sum test, Pearson’s Chi-squared test, and Fisher’s exact test were used as appropriate to compare MAKE ( +) group and MAKE (−) group

### Trajectory patterns

**Fig. 3:**
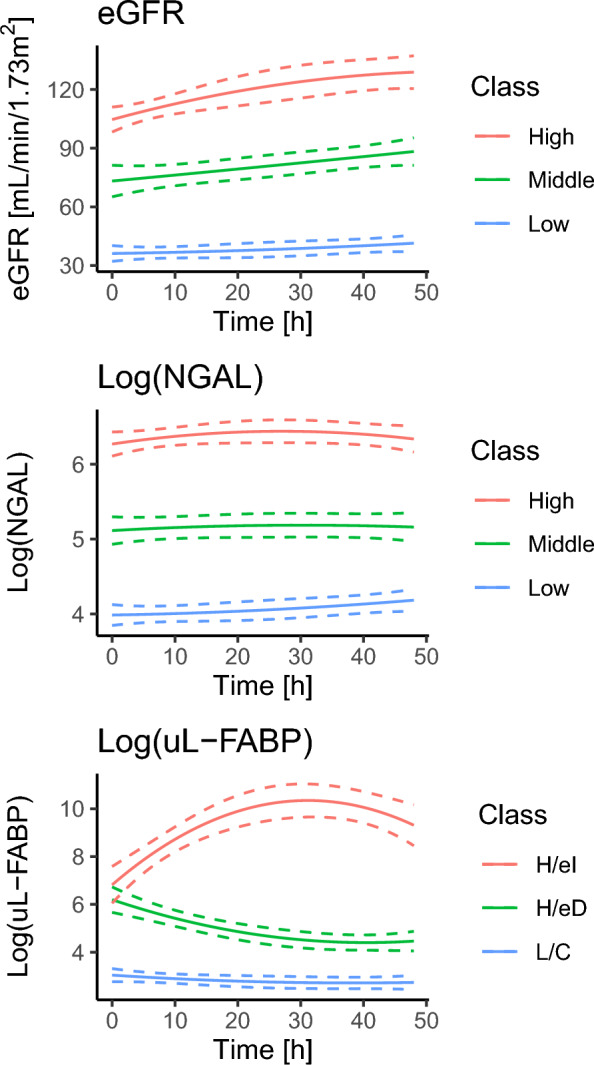
Trajectory patterns of kidney-related variables. Solid lines indicate the mean predicted trajectories. Dashed lines indicate the 95% confidence intervals. Note that NGAL and uL-FABP were log-transformed prior to the trajectory analysis. The trajectory models for eGFR and log (NGAL) showed almost parallel classes, with differences in magnitude only. In contrast, the model for log (uL-FABP) exhibited differences in both the magnitude and direction of changes. The class names of log (uL-FABP) are as follows. “High and exponential increase (H/eI),” “high and exponential decrease (H/eD),” and “low and constant (L/C).” *eGFR* estimated glomerular filtration rate, *uL-FABP* urinary liver-type fatty acid-binding protein, *NGAL* neutrophil gelatinase-associated lipocalin

As shown in Fig. 3, the trajectory models for eGFR and log (NGAL) showed almost parallel classes, with differences in magnitude only. In contrast, the model for log (uL-FABP) exhibited differences in both the magnitude and direction of changes. These classes were named “low and constant,” “high and exponential decrease,” and “high and exponential increase.” Table 2 summarizes the relationship between the classes for each variable and clinical outcome. In the eGFR and log (NGAL) models, patients in the higher severity classes for the variable of interest tended to show worse clinical outcomes. On the other hand, in the log (uL-FABP) model, patients in the “high and exponential increase” class had the worst clinical outcomes, followed by the “high and exponential decrease” and “low and constant” classes. Specifically, MAKE occurred in 18% (16/89), 40% (16/40), and 100% (9/9) of patients in the “low and constant,” “high and exponential decrease,” and “high and exponential increase” classes of the uL-FABP trajectory groups, respectively (p < 0.001). The temporal profile of AKI severity by uL-FABP trajectory classes is shown in supplemental Fig. 2. The patients in the “low and constant” group tended to have low AKI severity with minimal progression. The “high and exponential decrease” class included the patients with various levels of AKI severity and seemed to show the most remarkable tendency of recovery. AKI severity of the “high and exponential increase” class was the highest, with almost no recovery. Supplemental Fig. 3 is comparison of trajectory classes between different biomarkers. As expected, there appear to be associations in the trajectory patterns among these biomarkers. However, notably, many individuals in the “low” eGFR class were still in the “low and constant” uL-FABP class simultaneously.

**Table 2:** Trajectory patterns and clinical outcomes

eGFR, N = 144				
Outcome	Low, n = 72	Middle, n = 42	High, n = 30	p-value
MAKE	34 (47)	4 (10)	5 (17)	< 0.001
Day 7 AKI Stage^a^				< 0.001
No AKI	33 (46)	38 (90)	28 (93)	
Stage 1	16 (22)	2 (5)	2 (7)	
Stage 2	7 (10)	1 (2)	0 (0)	
Stage 3	11 (15)	1 (2)	0 (0)	
Day 1–7 AKI Stage change^a^				< 0.001
Improved	25 (35)	9 (21)	5 (17)	
Unchanged	27 (38)	32 (76)	24 (80)	
Deteriorated	15 (21)	1 (2)	1 (3)	
Death	18 (25)	1 (2)	3 (10)	0.002
RRT	16 (22)	1 (2)	0 (0)	< 0.001

Log (NGAL), N = 142				
Outcome	Low, n = 55	Middle, n = 50	High, n = 37	p-value
MAKE	10 (18)	15 (30)	18 (49)	0.009
Day 7 AKI Stage^a^				< 0.001
No AKI	48 (87)	35 (70)	17 (46)	
Stage 1	5 (9)	9 (18)	5 (14)	
Stage 2	1 (2)	3 (6)	3 (8)	
Stage 3	1 (2)	0 (0)	10 (27)	
Day 1–7 AKI Stage change^a^				0.008
Improved	11 (20)	14 (28)	14 (38)	
Unchanged	41 (75)	28 (56)	13 (35)	
Deteriorated	3 (5)	5 (10)	8 (22)	
Death	4 (7)	7 (14)	10 (27)	0.039
RRT	1 (2)	4 (8)	10 (27)	< 0.001

Log (uL-FABP), N = 138				
Outcome	Low & constant, n = 89	High & exp decrease^†^, n = 40	High & exp increase^†^, n = 9	p-value
MAKE	16 (18)	16 (40)	9 (100)	< 0.001
Day 7 AKI Stage^a^				< 0.001
No AKI	69 (78)	28 (70)	1 (11)	
Stage 1	13 (15)	5 (13)	1 (11)	
Stage 2	4 (4)	2 (5)	1 (11)	
Stage 3	2 (2)	4 (10)	4 (44)	
Day 1–7 AKI Stage change^a^				0.09
Improved	20 (22)	16 (40)	2 (22)	
Unchanged	60 (67)	17 (43)	4 (44)	
Deteriorated	8 (9)	6 (15)	1 (11)	
Death	8 (9)	6 (15)	6 (67)	< 0.001
RRT	3 (3)	4 (10)	6 (67)	< 0.001

NGAL and uL-FABP were log-transformed prior to the analysis

*MAKE* major adverse kidney events at hospital discharge *AKI* acute kidney injury *RRT* renal replacement therapy *eGFR* estimated glomerular filtration rate *NGAL* neutrophil gelatinase-associated lipocalin *uL-FABP* urinary liver-type fatty acid-binding protein

^†^”High & exp decrease” stands for high and exponential decrease, “High and exp increase” stands for high and exponential increase

^a^For those who had died by day 7, AKI status on day 7 was not defined and was treated as missing value

### Multivariate logistic regression model

Table 3 summarizes the logistic regression models for different sets of predictor variables. Both time0 (initial value at time 0 h) and delta12 (change in the first 12 h) of log (uL-FABP) were significantly associated with MAKE, whereas time0 and delta12 of eGFR were not significantly associated with MAKE when included in the model with log (uL-FABP). In the models with time0 and delta12 of eGFR and log (NGAL), no significant association with MAKE was observed. Although the areas under the receiver operating characteristic curves were similar for models including eGFR and log (uL-FABP) as variables of interest (Models 0–2), Model 2 (the model including delta12 and time0) showed significant improvement compared with Models 0 and 1, based on the cNRI and IDI (Supplemental Table 1).

**Table 3:** Multivariate logistic regression models with initial values and 12-h changes for prediction of major adverse kidney events

Model		Variable	Coefficient	SE	p-value	OR (95% CI)
Models on: eGFR, log (uL-FABP) N = 140	Model 0	**eGFR**_**0**_	**− 0.014**	**0.006**	**0.03**	**0.98 (0.97–0.99)**
		ΔeGFR	**− **0.018	0.013	0.18	0.98 (0.96–1.01)
	Model 1	eGFR_0_	**− **0.009	0.007	0.20	0.99 (0.98–1.00)
		ΔeGFR	**− **0.015	0.013	0.27	0.99 (0.96–1.01)
		**Log (uL-FABP)**_**0**_	**0.220**	**0.094**	**0.02**	**1.25 (1.04–1.51)**
	Model 2	eGFR_0_	**− **0.004	0.006	0.48	1.00 (0.98–1.01)
		ΔeGFR	**− **0.015	0.013	0.25	0.99 (0.96–1.01)
		**Log (uL-FABP)**_**0**_	**0.373**	**0.114**	**0.001**	**1.45 (1.17–1.83)**
		**ΔLog (uL-FABP)**	**0.357**	**0.131**	**0.006**	**1.43 (1.12–1.88)**
Models on: eGFR, log (NGAL) N = 146	Model 0’	**eGFR**_**0**_	**− 0.014**	**0.006**	**0.03**	**0.98 (0.97–1.00)**
		ΔeGFR	**− **0.017	0.013	0.18	0.98 (0.96–1.01)
	Model 1’	eGFR_0_	**− **0.009	0.007	0.19	0.99 (0.98–1.00)
		ΔeGFR	**− **0.017	0.013	0.19	0.98 (0.96–1.01)
		Log (NGAL)_0_	0.255	0.214	0.23	1.29 (0.85–1.97)
	Model 2’	eGFR_0_	**− **0.008	0.007	0.22	0.99 (0.98–1.00)
		ΔeGFR	**− **0.009	0.013	0.48	0.99 (0.96–1.02)
		Log (NGAL)_0_	0.349	0.220	0.11	1.42 (0.92–2.20)
		ΔLog (NGAL)	0.822	0.463	0.08	2.28 (0.93–5.79)

All models were built with major adverse kidney events (MAKE) at hospital discharge as the outcome. Patients with missing values in the variables of interest were excluded from the analysis on those variables. OR was calculated for every 1 unit increase of each variable (for NGAL and uL-FABP, OR for an increase of the log-transformed value by 1 point). Bold values denote statistical significance

*SE* Standard error, *OR* Odds ratio, *CI* Confidence interval, *eGFR* estimated glomerular filtration rate, *NGAL* neutrophil gelatinase-associated lipocalin, *uL-FABP* urinary liver-type fatty acid-binding protein, *eGFR*_*0*_ initial value of eGFR, *ΔeGFR* 12-h change of eGFR, *Log (uL-FABP)*_*0*_ initial value of log (uL-FABP), *ΔLog (uL-FABP)* 12-h change of log (uL-FABP), *Log (NGAL)*_*0*_ initial value of log (NGAL), *ΔLog (NGAL)* 12-h change of log (NGAL)

Based on these results, FABPi was defined as the fitted value for Model 2. FABPi was calculated using the covariates in Model 2, as shown in the following formula:

$$FABPi= \frac{exp[-2.144 - 0.004\times {eGFR}_{0} - 0.015\times \Delta eGFR + 0.373\times {Log\left(uL-FABP\right)}_{0} + 0.357\times \Delta Log\left(uL-FABP\right)]}{1+exp[-2.144 - 0.004\times {eGFR}_{0} - 0.015\times \Delta eGFR + 0.373\times {Log\left(uL-FABP\right)}_{0} + 0.357\times \Delta Log\left(uL-FABP\right)]}$$

where eGFR_0_ stands for the initial eGFR value, ΔeGFR stands for the 12-h change in eGFR, log (uL-FABP)_0_ stands for the initial value of log (uL-FABP), and Δlog (uL-FABP) stands for the 12-h change in log (uL-FABP).

The best cutoff of FABPi to predict MAKE was calculated as 0.299 with sensitivity of 0.585 and specificity of 0.778, using Youden index (Supplemental Fig. 4). Supplemental Table 2 shows the clinical characteristics likely to influence MAKE (i.e., potential confounders) before and after balancing using IPW. The standard mean difference for each variable was less than 0.1 in the weighted patient data, suggesting an appropriate balancing. The odds ratio of positive FABPi for MAKE with a 95% confidence interval was calculated as 3.74 (1.52–9.19) (p = 0.004).

## Discussion

In this prospective observational study with serial measurements, the chronological changes (i.e., trajectory) of AKI biomarkers and eGFR were clustered into three subgroups and visualized using group-based trajectory modeling. In one study, this statistical method was used to categorize the trajectory patterns of kidney function over a longer period of years, in the setting of an epidemiological research on chronic kidney health [35]. To the best of our knowledge, this is the first study to evaluate the trajectory patterns of AKI biomarkers in ICU patients, in acute settings. The trajectory subcategories showed significant associations with clinical outcomes for eGFR, log (NGAL), and log (uL-FABP); lower eGFR, higher NGAL, and higher uL-FABP were associated with worse outcomes (Fig. 3 and Table 2). These findings are concordant with previous studies showing the association of severity in these renal variables and clinical outcomes [13, 15–24, 36, 37].

A unique finding of this study was the differences in the shape of the trajectory patterns for log (uL-FABP) compared to eGFR and log (NGAL) (Fig. 3). Both the magnitude and the direction of the chronological changes constituted important characteristics of the trajectory for log (uL-FABP). The association of the unique trajectory patterns for log (uL-FABP) with poor clinical outcomes was demonstrated in a stepwise manner from “low and constant” to “high and exponential increase” (Table 2). In contrast, the trajectory curves of eGFR and log (NGAL) paralleled each other, meaning that the magnitude relationship in eGFR and log (NGAL) did not change during the initial 48 h after ICU admission. These findings suggest that identifying the uL-FABP trajectory pattern by serial measurements may give additional information during the early hours after ICU admission. This may be because uL-FABP reflected tubular damage more accurately than the other two indices. A sustained increase in tubular damage biomarkers might reflect ongoing persistent kidney injury, which add more information on the undergoing pathological process over time, leading to MAKE events. This assumed mechanism supports the result that “high and exponential decrease” uL-FABP trajectory class tended to show higher rate of recovery in AKI severity (supplemental Fig. 2). There seem discrepancies among the trajectory classes of eGFR, log (NGAL), and log (uL-FABP) (supplemental Fig. 3). This may be because uL-FABP was able to reflect kidney damage better than the others. Estimated GFR reflected not kidney damage but kidney function, whereas NGAL in blood might fail to reflect kidney damage.

Another important finding of this study is the integrated model consisting of two different variables for temporal changes in uL-FABP, i.e., time0 and delta 12 (Table 3). Time0 and delta12 of log (uL-FABP) were significantly associated with MAKE after adjusting for eGFR, whereas time0 and delta12 of log (NGAL) did not significantly associate with MAKE. This suggests that both the initial value (i.e., initial severity) and the early chronological changes (i.e., trend) of log (uL-FABP) may independently impact clinical outcomes, highlighting the importance of trajectory analysis. In one study, patients with increased uL-FABP levels (measured using a semi-quantitative kit) during the initial 6 h after ICU admission had higher mortality rates compared with the mortality rates in patients with decreased or unchanged uL-FABP levels [23]. In addition, the binary index (i.e., positive or negative FABPi), based on the best model according to the cNRI and IDI (Supplemental Table 1), significantly associated with MAKE with a high OR [3.74 (1.52–9.19)], even after balancing for potential confounders (Supplemental Table 2). In acute clinical settings, such as emergency departments, the acuteness of renal dysfunction is often difficult to determine [38] and an information on acute renal damage would be more helpful to make a clinical judgement. Based on the findings of our study, serial uL-FABP measurements in the early clinical course may be helpful in such acute clinical settings.

In this study, biomarkers were measured at time 0, 12, 24, and 48 h after ICU admission. However, the most appropriate timing and frequency of measurements has not been determined. Although more frequent measurements would give more accuracy, this may not be feasible due to an increased medical cost for many measurements. Previous studies showed that uL-FABP may peak within 6 h of cardiac surgery and continue to change for several days [39, 40]. However, in general ICU patients, the peak timing would be less clear than in surgical patients. In addition to the frequency of measurements, the duration of intra-measurements is another issue. Evaluation of changes with longer observation period may enable to predict long term outcomes such as MAKE more accurately.

An observational study reported that changes in plasma NGAL levels during the first 48 h predicted mortality in 50 ICU patients with AKI stage 2 or higher [41]. Another observational study showed that NGAL declined more between the 24–48-h and 5–7-day measurements after ICU admission in patients without MAKE at discharge compared with the patients who developed MAKE [42]. In our study, time0 and delta12 of log (NGAL) failed to show a significant association with MAKE in the multivariate logistic regression models (Table 3). In addition to differences in the biological characteristics of NGAL and L-FABP [9, 43], the optimal timeframe of serial measurements may be different. It is also important to consider the difference between plasma and urine samples. The different findings of NGAL and uL-FABP in our study might be attributable to the difference between plasma and urine, rather than NGAL and L-FABP. It would be one of the interesting future steps, to investigate the clinical characteristics of urinary NGAL and its trajectory patterns. Further evaluation is necessary to determine the best measurement strategy for these biomarkers.

There are several limitations to this study. First, this is a single-center study and the population may have been biased. In addition, enrollment in this study was limited to patients with an arterial line and a urinary catheter. Although these are common interventions in the ICU, similar findings may not be observed in less severely ill patients without these devices. Thus, caution should be applied when generalizing the results of this study. Second, due to the small sample size, trajectory modeling might miss small trajectory classes and the lack of validation cohort might lead to an overadjustment. Although the confounders in this study were pre-specified, the final sample size was not sufficiently large for a simple multivariate logistic regression analysis with all confounders in one model. Hence, combination of logistic regression and inverse probability weighting was selected in this study. These might be complicated processes and could be a source of overadjustment as well. Third, the number of classes in the trajectory analysis was determined by both mathematical indices such as Bayesian information criterion but also by model interpretability; thus, this method might include some arbitrariness. In addition, it should be noted that there are no established methods to adjust the potential confounders within the trajectory analysis. Although the logistic regression model results are concordant with the results of the trajectory analysis, the overall findings of this study might be influenced by such confounders to some extent. The statistical methods used in this study may have several more limitation. Youden index gives the most “balanced” cutoffs, assuming sensitivity and specificity are equally important. This assumption might not always be true. The interpretation of cNRI is not intuitive and the model selection may be influenced by overfitting. The patients with missing values had to be excluded from the analyses in both the group-based trajectory modeling and logistic regression models. The common reasons for missing values were removal of the arterial lines or the urinary catheter during the study and anuric status, making blood or urine sample collection impossible. However, even in the model with the highest number of excluded patients, 88% of patients were still available for analysis [trajectory model of log (uL-FABP), 138 out of 156 patients]. Lastly, AKI was diagnosed only by sCr in this study. Although the KDIGO AKI diagnostic criteria include UOP information, the clinical indication of sCr and UOP may not be interchangeable. The sCr-based AKI stage was sometimes different from the UOP-based stage, even in the same patient [44]. In addition, one study showed that patients with UOP-based AKI stage 3 had a higher mortality rate than sCr-based AKI stage 3 [45]. In this study, anuric patients were excluded from the analysis of uL-FABP; thus, extra caution should be applied when interpreting the results.

## Conclusions

The trajectory patterns of AKI biomarkers, especially the patterns of uL-FABP changes were significantly associated with MAKE, indicating the importance of the biomarker trajectory in ICU practice. The addition of chronological changes to the initial severity of uL-FABP may contribute to discriminate worsening critically ill patients from others. Further investigation is warranted to develop the clinical management strategy that utilizes biomarker trajectories.

### Supplementary Information

Supplementary Material 1.

Supplementary Material 2.

Supplementary Material 3.

## Data Availability

The dataset supporting the conclusions of this article is included within the article (and its additional file (s)).

## Abbreviations

AKI: Acute kidney injury
ICU: Intensive care unit
RRT: Renal replacement therapy
CKD: Chronic kidney disease
KDIGO: Kidney Disease: Improving Global Outcomes
sCr: Serum creatinine
UOP: Urine output
NGAL: Neutrophil gelatinase-associated lipocalin
L-FABP: Liver-type fatty acid-binding protein
uL-FABP: Urine L-FABP
GBTM: Group-based trajectory modeling
MAKE: Major adverse kidney event
ESRD: End-stage renal disease
eGFR: Estimated glomerular filtration rate
MDRD: Modified Diet Renal Disease
cNRI: Continuous net reclassification improvement
IDI: Integrated discrimination improvement
FABPi: Integrated uL-FABP index
APACHE II: Acute physiology and chronic health evaluation II
IPW: Inverse probability weighting
SOFA: Sequential organ failure assessment
