# Supplementary material for: Trajectory pattern of serially measured acute kidney injury biomarkers in critically ill patients: a prospective observational study
Source: Ann Intensive Care. 2024 Jun 6;14:84. doi: 10.1186/s13613-024-01328-9 (PMC11156822; doi:10.1186/s13613-024-01328-9)
Supplement: Supplementary file 1 — Supplementary Material 1. [file 13613_2024_1328_MOESM1_ESM.docx]

**Supplemental document. Overview of the trajectory analysis in this study**

**Statistical details**

In this study, a statistical method called “group-based trajectory modeling (GBTM)” was used to analyze the trajectory patterns. GBTM allows to classify a group of subjects into a finite number of subgroups with common trajectory patterns of a continuously changing variable over time(1). This method can be performed in various platforms, including SAS software and R software, by using appropriate packages. One of those packages for R software (version 4.1.0 was used in this study) is lcmm package (version 1.9.4 was used in this study)(2).

The lcmm package can perform a statistical method called “latent class trajectory modeling,” which is more generalized form of GBTM, and generally allows within-class variability, i.e., random effects. GBTM can be run as a special form of latent class trajectory modeling, where there are no random effects. An example of latent class trajectory modeling is given by the following formula.

$$Y_{itk}=\left( \beta_{0}^{k}+b_{0}^{k} \right)+\left( \beta_{1}^{k}+b_{1}^{k} \right){Time}_{t}+{(\beta}_{2}^{k}+b_{2}^{k}){Time}_{t}^{2}+\varepsilon_{t}$$

Where *Y* is a variable of interest [such as eGFR, log(NGAL) or log(uL-FABP)] of individual *i* = 1, …, N, at time *t* = 1, …, T, in class *k* = 1, …, K, and *b* is random effects (Within-class variability. Class-specific and follows multivariate Normal distribution with zero mean and a 3×3 variance-covariance matrix *B*). ε represents a residual error which is normally distributed with zero mean and variance σ^2^. In lcmm package, by giving the number of classes K, the β’s above are determined based on the data given(3).

Since GBTM does not include random effects(1, 2), lcmm package in the current study was used under the setting of no random effects. Hence, the models can be described as follows.

$$Y_{itk}=\beta_{0}^{k}+\beta_{1}^{k}{Time}_{t}+\beta_{2}^{k}{Time}_{t}^{2}+\varepsilon_{t}$$

In this study, the values of the biomarkers (*Y* in the formula above) were assumed to follow the quadratic function of time. This was to express up to one peak within 48 hours of serial measurements.

When performing GBTM, the number of classes must be given. To determine the most appropriate number of classes K, multiple points need to be considered. In this study, the following were considered.

- The models should fit the data well, but should be useful and parsimonious (The model has a good interpretability).
- Bayesian information criteria is sufficiently low and difference of it between two models with different numbers of trajectory subgroups is small enough.
- Each subclass has sufficient number of individuals (for example, ≧3% of the whole patients).
- Average of posterior probability assignment≧0.7.
- Relative entropy (expressed as 0 to 1) is close to 1.

**The actual process of the model determination**

In the current study, the trajectory patterns for eGFR, log(NGAL), and log(uL-FABP) were analyzed. For the trajectory of eGFR, one outlier exhibited extraordinarily high eGFR values that could not be included in any trajectory class; therefore, this patient was excluded from the analysis (Figure 1). As shown in Supplemental figure D1, relatively remarkable reductions in Bayesian information criteria were observed when the number of trajectory classes increased up to 4. In contrast, reductions in Bayesian information criterion were less outstanding for further increases in the number of classes. The performances of the 3-class and 4-class models are summarized in Supplemental table D1. Both model performances were acceptable. Therefore, the 3-class and 4-class models were compared (Figure 3 and Supplemental figure D2). The 4-class and 3-class models exhibited similar patterns, with only one additional trajectory class parallel to the adjacent one, representing an additional layer of severity. From the model interpretability standpoint, 3-class models were simpler, easier to interpret, and sufficient to describe the characteristics of the population. Therefore, 3-class models were used for further analyses.

**References**

1. Nagin DS, Odgers CL. Group-based trajectory modeling in clinical research. Annu Rev Clin Psychol. 2010;6:109-38.

2. Nguena Nguefack HL, Pagé MG, Katz J, Choinière M, Vanasse A, Dorais M, et al. Trajectory Modelling Techniques Useful to Epidemiological Research: A Comparative Narrative Review of Approaches. Clin Epidemiol. 2020;12:1205-22.

3. Lennon H, Kelly S, Sperrin M, Buchan I, Cross AJ, Leitzmann M, et al. Framework to construct and interpret latent class trajectory modelling. BMJ Open. 2018;8(7):e020683.


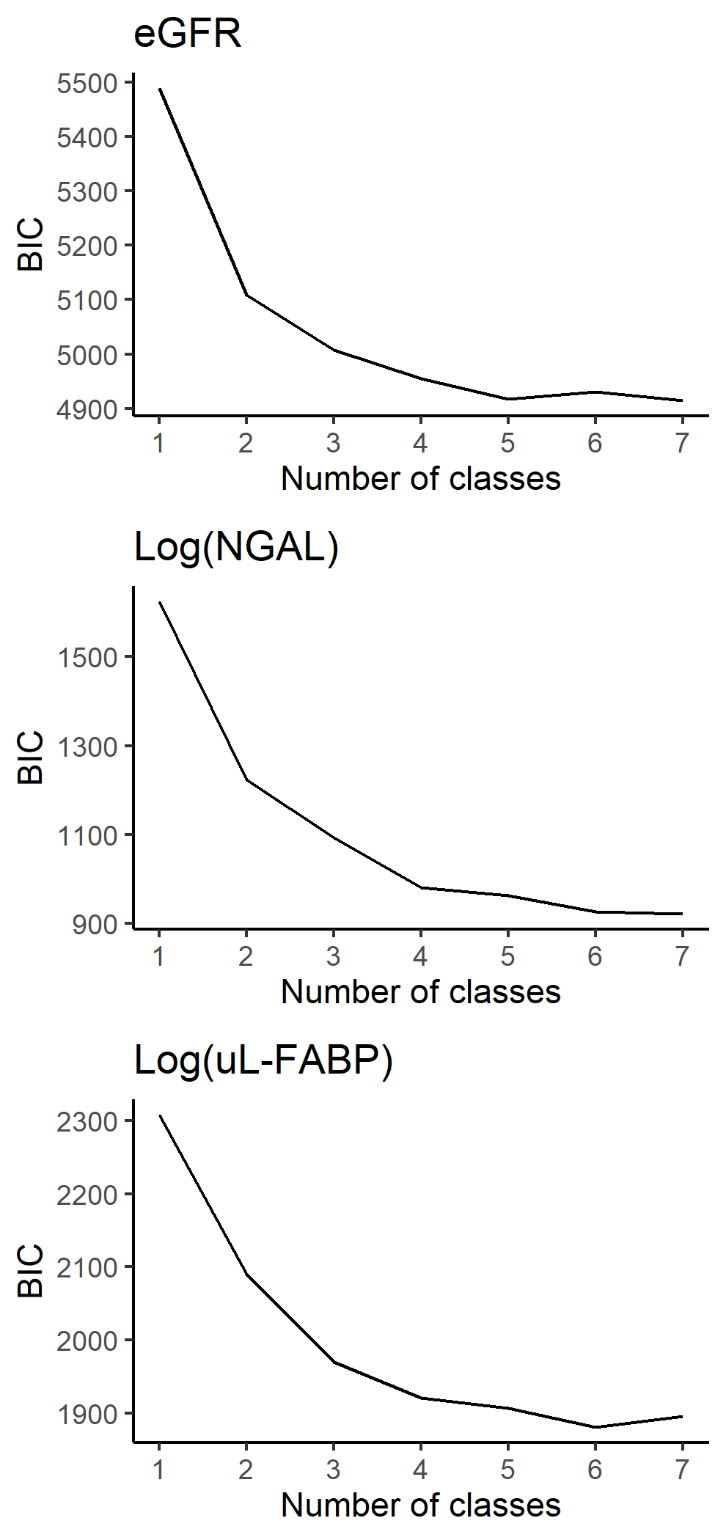


**Supplemental figure D1. Elbow plots for group-based trajectory modeling**

*BIC*, Bayesian information criterion; *eGFR*, estimated glomerular filtration rate; *uL-FABP*, urinary liver-type fatty acid-binding protein; *NGAL*, neutrophil gelatinase-associated lipocalin.

**Supplemental table D1. Summary of group-based trajectory modeling performances**

| Variable | Number of classes | Patients analyzed | BIC | APPA | | | | Number of patients assigned in the class | | | | Relative entropy |
| --- | --- | --- | --- | --- | --- | --- | --- | --- | --- | --- | --- | --- |
|  |  |  |  | Class 1 | Class 2 | Class 3 | Class 4 | Class 1 | Class 2 | Class 3 | Class 4 |  |
| eGFR | 3 | 144 | 5006.8 | 0.977 | 0.950 | 0.943 |  | 72(50) | 42(29) | 30(21) |  | 0.913 |
|  | 4 | 144 | 4954.7 | 0.892 | 0.915 | 0.954 | 0.923 | 30(21) | 51(35) | 37(26) | 26(18) | 0.875 |
| Log(NGAL) | 3 | 142 | 1093.7 | 0.968 | 0.950 | 0.961 |  | 55(39) | 50(35) | 37(26) |  | 0.906 |
|  | 4 | 142 | 982.0 | 0.973 | 0.953 | 0.966 | 0.965 | 32(23) | 48(34) | 40(28) | 22(15) | 0.935 |
| Log(uL-FABP) | 3 | 138 | 1970.2 | 0.966 | 0.936 | 0.998 |  | 89(64) | 40(29) | 9(7) |  | 0.906 |
|  | 4 | 138 | 1920.2 | 0.933 | 0.896 | 0.979 | 0.999 | 47(34) | 63(46) | 19(14) | 9(7) | 0.866 |

*BIC*, Bayesian information criterion; *APPA*, average of posterior probability assignment; *eGFR*, estimated glomerular filtration rate; *NGAL*, neutrophil gelatinase-associated lipocalin; *uL-FABP*, urinary liver-type fatty acid-binding protein.

The performances of the 3 or 4-class models are shown.


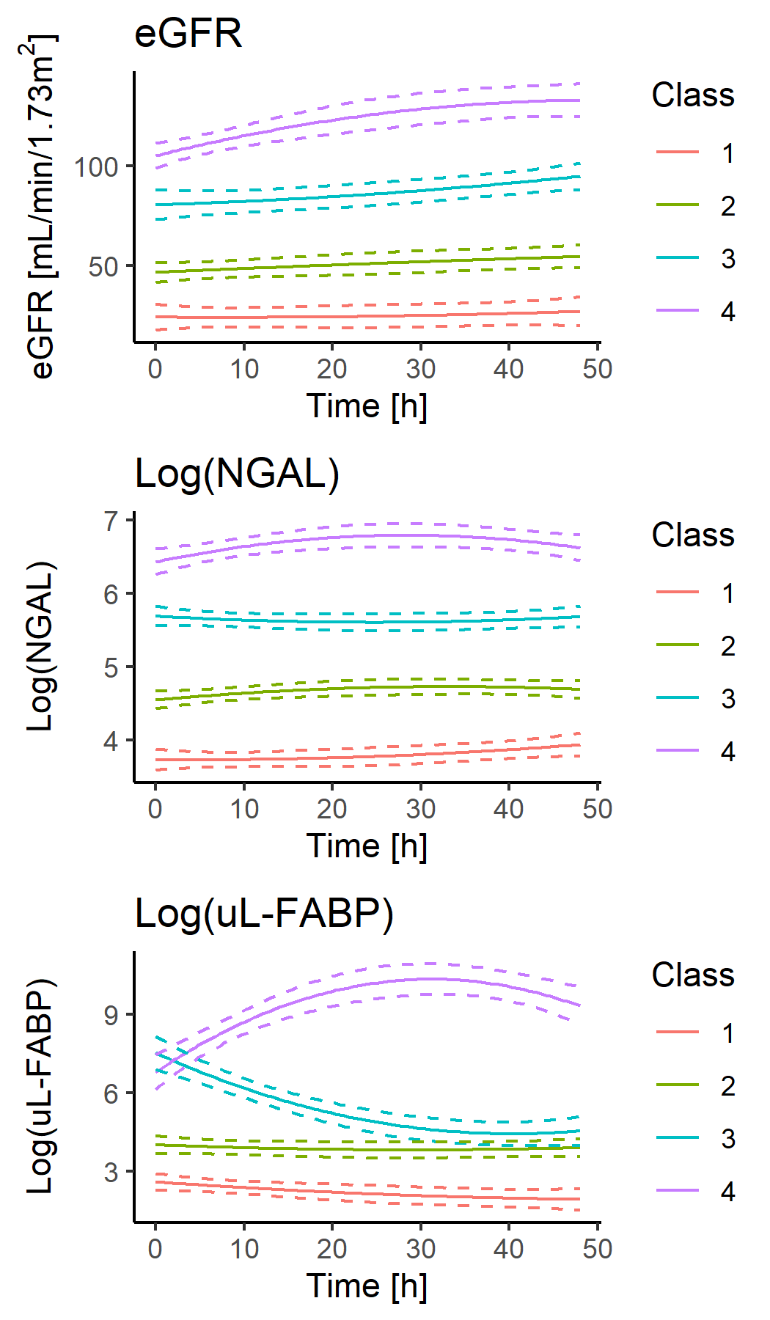


**Supplemental figure D2. Trajectory patterns of kidney-related variables (4-class models)**

Solid lines indicate the mean predicted trajectories. Dashed lines indicate the 95% confidence intervals.

*eGFR*, estimated glomerular filtration rate; *uL-FABP*, urinary liver-type fatty acid-binding protein; *NGAL*, neutrophil gelatinase-associated lipocalin.
