# Supplementary material for: Trajectory pattern of serially measured acute kidney injury biomarkers in critically ill patients: a prospective observational study
Source: Ann Intensive Care. 2024 Jun 6;14:84. doi: 10.1186/s13613-024-01328-9 (PMC11156822; doi:10.1186/s13613-024-01328-9)
Supplement: Supplementary file 2 — Supplementary Material 2. [file 13613_2024_1328_MOESM2_ESM.docx]

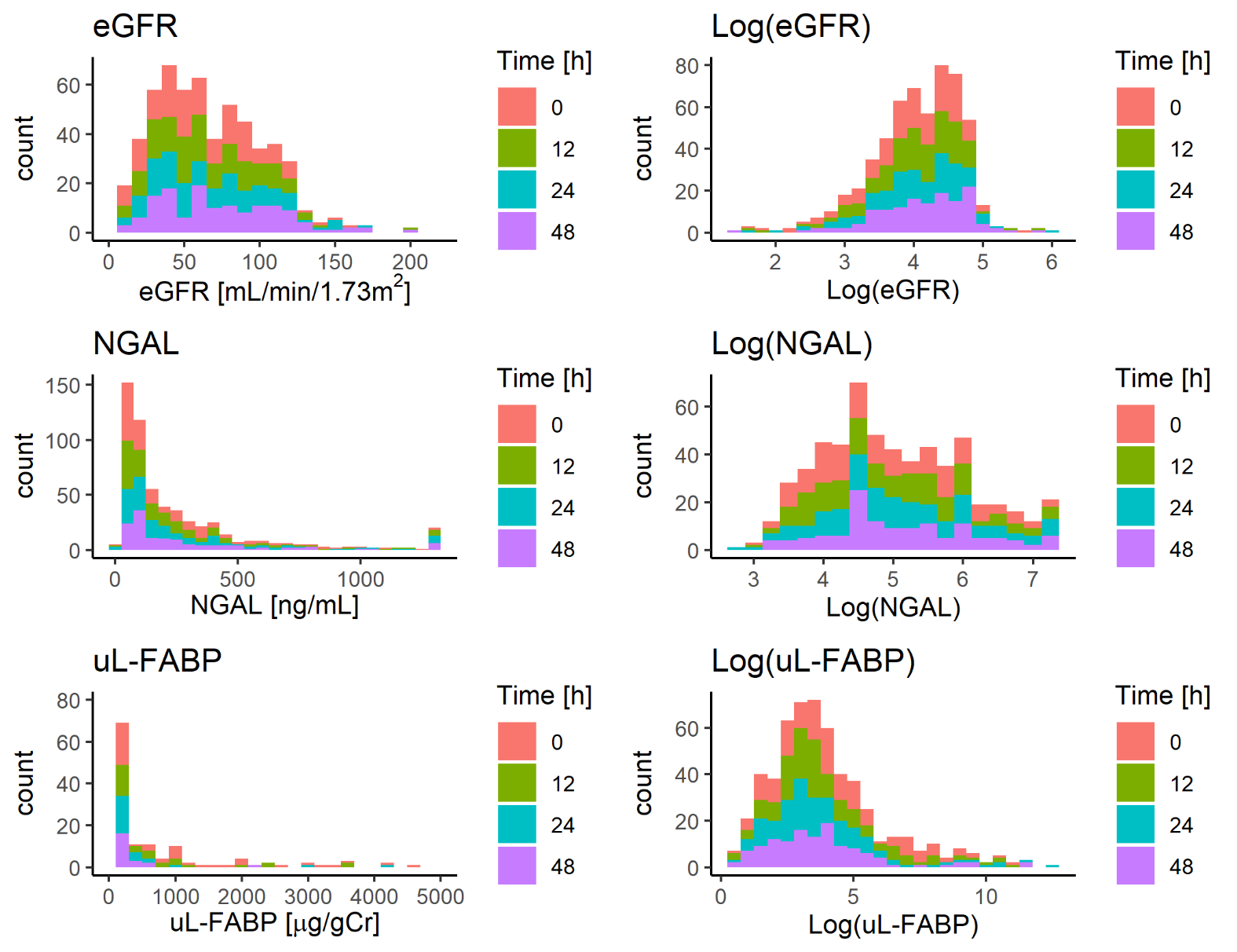


**Supplemental figure 1. Distributions of kidney-related variables**

*eGFR*, estimated glomerular filtration rate; *uL-FABP*, urinary liver-type fatty acid-binding protein; *NGAL*, neutrophil gelatinase-associated lipocalin.

**Supplemental figure 2. Temporal profile of AKI severity by uL-FABP trajectory classes**

*AKI*, acute kidney injury; *uL-FABP*, urinary liver-type fatty acid-binding protein.

The patients in the “low and constant” group tended to have low AKI severity with minimal progression. The “high and exponential decrease” class included the patients with various level of AKI severity and seemed to show the most remarkable tendency of recovery. AKI severity of the “high and exponential increase” class was the highest, with almost no recovery.

**Supplemental figure 3. Comparison between the biomarker trajectory subclasses**

*eGFR*, estimated glomerular filtration rate; *uL-FABP*, urinary liver-type fatty acid-binding protein; *NGAL*, neutrophil gelatinase-associated lipocalin.

Note that NGAL and uL-FABP were analyzed and classified after log-transformation.

*The class names of log(uL-FABP) are as follows. High and exponential increase (H/eI), high and exponential decrease (H/eD), and low and constant (L/C).

**Supplemental table 1. Comparison of multivariate logistic regression models**

| Model | Variables | AUROC | cNRI | IDI | Compared with |
| --- | --- | --- | --- | --- | --- |
| Model 0 | eGFR_0_, ΔeGFR | 0.72 (0.62 – 0.83) | NA | NA |  |
| Model 1 | eGFR_0_, ΔeGFR, Log(uL-FABP)_0_ | 0.71 (0.61 – 0.80) | 0.34 (-0.02 – 0.70) | 0.028 (-0.008 – 0.065) | Model 0 |
| Model 2 | eGFR_0_, ΔeGFR, Log(uL-FABP)_0_, ΔLog(uL-FABP) | 0.71 (0.62 – 0.81) | **0.53 (0.18 – 0.88)** | **0.087 (0.020 – 0.154)** | Model 0 |
|  |  |  | **0.42 (0.07 – 0.78)** | **0.059 (0.004 – 0.114)** | Model 1 |

*AUROC*, area under receiver operating characteristic curve; *cNRI*, continuous net reclassification improvement; *IDI*, integrated discrimination improvement; *eGFR*, estimated glomerular filtration rate; *uL-FABP*, urinary liver-type fatty acid-binding protein.

AUROC was calculated for prediction of major adverse kidney events (MAKE) at hospital discharge. eGFR_0_ and Log(uL-FABP)_0_ stand for the values at time 0h (time0). ΔeGFR and ΔLog(uL-FABP) stand for the change between time 0h and time 12h (delta12).


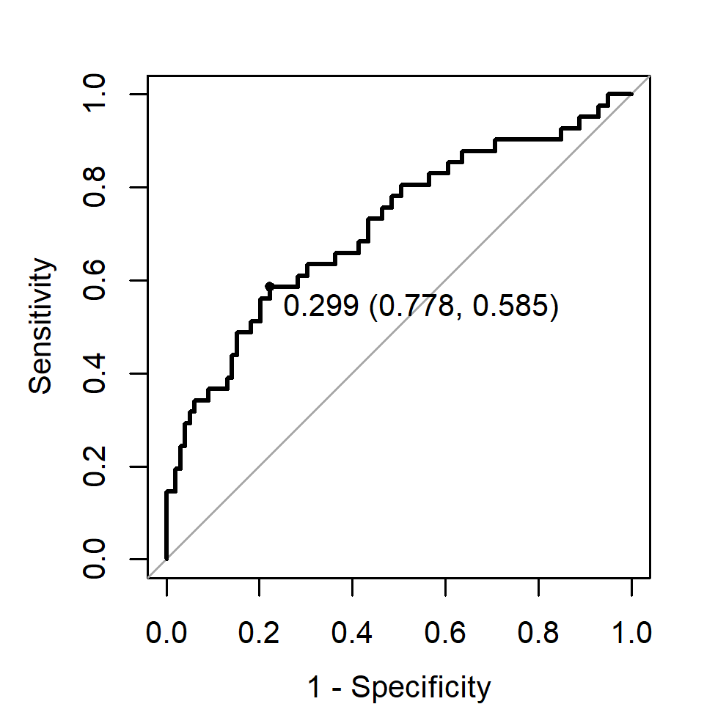


**Supplemental figure 4. Receiver operating characteristic curve of integrated uL-FABP index to predict major adverse kidney events**

*uL-FABP*, urinary liver-type fatty acid-binding protein.

The number shows the cutoff value of uL-FABP index (0.299) to predict major adverse kidney events at discharge, with the specificity and sensitivity calculated as 0.778 and 0.585 respectively.

**Supplemental table 2. Key characteristics before and after inverse probability weighting**

|  | Crude | | | Weighted | | |
| --- | --- | --- | --- | --- | --- | --- |
|  | Integrated uL-FABP index | | SMD | Integrated uL-FABP index | | SMD |
|  | Negative | Positive |  | Negative | Positive |  |
| n | 94 | 46 |  | 142.8 | 135.5 |  |
| Male | 57 (60.6) | 29 (63.0) | 0.05 | 87.5 (61.3) | 86.6 (63.9) | 0.055 |
| Age | 60.34 (16.7) | 67.28 (17.3) | 0.408 | 62.72 (15.8) | 63.79 (18.0) | 0.064 |
| Surgical | 51 (54.3) | 13 (28.3) | 0.547 | 62.9 (44.0) | 57.7 (42.6) | 0.03 |
| APACHE II | 15.66 (6.36) | 19.17 (6.95) | 0.528 | 16.61 (6.45) | 17.11 (6.48) | 0.078 |
| CKD | 18 (19.1) | 13 (28.3) | 0.216 | 38.6 (27.1) | 35.6 (26.3) | 0.018 |
| Sepsis | 4 (4.3) | 13 (28.3) | 0.688 | 20.3 (14.2) | 17.4 (12.8) | 0.041 |
| Shock | 6 (6.4) | 14 (30.4) | 0.653 | 22.9 (16.0) | 19.2 (14.2) | 0.052 |
| UTI | 1 (1.1) | 5 (10.9) | 0.423 | 8.2 (5.7) | 6.2 (4.6) | 0.053 |

*uL-FABP*, urinary liver-type fatty acid-binding protein; *SMD*, Standardized Mean Difference; *APACHE II*, acute physiology and chronic health evaluation II score; *CKD*, chronic kidney disease; *UTI*, urinary tract infection. Continuous variables are shown in mean (standard deviation). Categorical variables are shown in count (percentage).
